# The Effects of Mental Stress on Non-insulin-dependent Diabetes: Determining the Relationship Between Catecholamine and Adrenergic Signals from Stress, Anxiety, and Depression on the Physiological Changes in the Pancreatic Hormone Secretion

**DOI:** 10.7759/cureus.5474

**Published:** 2019-08-24

**Authors:** Hilda Wong, Jaya Singh, Ryan M Go, Nancy Ahluwalia, Michelle A Guerrero-Go

**Affiliations:** 1 Primary Care, California Institute of Behavioral Neurosciences and Psychology, Fairfield, USA; 2 Internal Medicine, Avalon University School of Medicine, Curcacao, USA; 3 Primary Care, California Instititute of Behavioral Neurosciences and Psychology, Fairfield, USA

**Keywords:** diabetes, anxiety, depression, type 1 diabetes, type 2 diabetes, diabetes mellitus, epinephrine, adrenaline, diabetes education, primary care

## Abstract

Non-insulin-dependent diabetes or type II diabetes is prevalent around the world. A high-fat diet and chronic inactivity are often responsible for this chronic ailment. However, it is suspected that a high level of stress can also exacerbate diabetes. High anxiety can result in the release of sympathetic hormones that can elevate both cortisol and glucose levels, decrease insulin release, or affect the sensitivity and resistant of the insulin hormone. We have analyzed three research articles to see how stress and anxiety can affect non-insulin-dependent diabetes. In the first article, we selected participants with type II diabetes and injected them with saline or norepinephrine. The results indicated that participants with norepinephrine had experienced a decrease in glucose disposal and reduction in insulin secretion rate. Our second article utilizes African-American adults with type II diabetes. We provide them with a survey to determine how stress, anxiety, and depression can affect adherence to lifestyle modifications such as exercise and eating a proper diet. We find that subjects with higher stress levels tend to have lower compliance with their lifestyle regimes. Our third article focuses on female participants and divides them into two categories which are high chronic stress (HCS) and low chronic stress (LCS). We use an MRI to observe their brain activity while they stare at a picture of high-caloric type food. Our results indicate that there are different responses in various brain structure activities between subjects with HCS and LCS group. With these analyses, it can improve on the way healthcare providers can consult with their patients who have exacerbated type II diabetes despite proper medication and lifestyle modification.

## Introduction and background

It can be frustrating for diabetic patients who have experienced hyperglycemia despite lifestyle modifications. Because of the results, physicians would enforce stricter weight loss or dieting regime without realizing that other underlying factors could be the culprit of these unfortunate results [1]. Keep in mind, that a lack of nutrition and constant starvation can only aggravate diabetes, which is a widespread issue that happens to a lot of patients [2]. Diabetes is a challenging illness to live with, and often times the proper lifestyle modification is just not enough. The typical solution that doctors usually opt for is increasing the dosage of metformin or insulin. Unfortunately, these medications can lead to unfavorable side effects such as lactic acidosis, gastrointestinal issues, and hypoglycemia [3]. What doctors do not know is that everyday stress and anxiety from life can affect the patient's insulin and glucose function, which can exacerbate their diabetes. Plus, anxiety in people without diabetes can put them at risk of weight gain and high cholesterol which can eventually lead them to hyperglycemia. 

The regular physiological function of the pancreas is to produce insulin that will bring glucose from the blood and back to the liver for storage [4]. The pancreas can produce insulin when it detects too much glucose in the blood, which can help prevent potential hyperglycemia [5]. However, people with type 1 diabetes are born to have a mutation where they are not able to produce adequate amounts of insulin [6]. Type II diabetes or non-insulin-dependent diabetes, on the other hand, is where the individual has absorbed a massive amount of calories daily without any proper exercising or dieting regime [7]. This constant high glucose levels can damage the insulin sensory system. Therefore, with non-insulin-dependent diabetes, the insulin does not respond to the high glucose level [7]. When that happens, the individual will eventually develop type II diabetes. When patients go through physiological, mental, or pathological stress, they will release adrenergic and catecholamine hormones which are norepinephrine and epinephrine from the adrenal medulla. The sympathetic hormone plays a significant role in affecting your blood sugar level and the function of your insulin. These adrenergic hormones can stimulate glucose production and reduce the insulin level, therefore worsening the diabetic condition [8].

Given our understanding of the adrenergic hormones and pathophysiology of type 2 diabetes mellitus, these chemical signals still have unpredictable results in our body. For example, the adrenergic hormones play a sympathetic role in our body, which is the fight or flight response. Because of the sympathetic functions, it would often suppress our appetite, therefore making us eat less [9]. For example, if a dinosaur were to chase you across the field, you will not stop by a McDonalds to get a hamburger, this would make no sense at all. Your adrenal gland will produce norepinephrine and epinephrine to suppress your appetite, make your heart beat faster, and improve your circulation and muscle strength to help you run faster [10]. This response is the same thing with stress and anxiety in a person's life. If you are always working in a job with a tight deadline or you are a police officer out in the field protecting civilians, your adrenalin is often in the upper level. Then this brings us to the question of why do some people lose a significant amount of weight while others are gaining weight during a stressful situation? Another critical issue that we should focus on is if adrenergic hormones play a role to suppress your appetite then why does it reduce insulin sensitivity? If these hormones suppress your appetite, that means you will not be eating a lot; therefore, it should lower your blood sugar level and improve your insulin sensitivity. However, a lot of research is showing that adrenergic hormones are exacerbating diabetes. Therefore, these body signals are playing a critical contradictory function in our body.

 What we speculate based on years of research is not what we expect in real life. There are a lot of chemical signals and factors that make this chronic illness extremely unpredictable. This observation also goes into the concept of dieting and weight loss. People can be very disciplined with their diet and exercise but are not able to lose an adequate amount of weight; in fact, they gain weight instead. If we look at this in another perspective, we can see that everyday stress and anxiety such as family obligations, deadlines, work, drama, relationships, etc. can cause other chemical signals such as serotonin and endorphins to play a role in our weight, metabolism, and pancreatic function. Because of the multifaceted chemical signals that occur from stressful situations, it is essential that healthcare providers are aware of these factors, and can find strategic ways to help the patient to cope. That way, the patient will know how to properly manage their stress without causing a massive fluctuation of hormones in their body that can affect their progress towards a healthier lifestyle. Once we keep these hormones under control, then patients would be able to manage their weight and diabetes more efficiently.

## Review

Non-insulin-dependent diabetes is the main focus for this research because type II diabetes involves increased insulin resistance, which can be developed over time [11]. Individuals usually do not get type II diabetes through birth, but they acquire it through horrible dieting, constant inactivity, and other illnesses such as high cholesterol or hypothyroidism [12]. However, constant stress that stimulates the release of catecholamine and adrenergic hormone can also play a role in exacerbating type II diabetes [13]. There are a variety of clinical research studies out there that showcase the effects of stress and anxiety in type II diabetes.

The effect of norepinephrine on insulin secretion and glucose effectiveness in non-insulin-dependent diabetes

This research utilizes eight subjects with type II diabetes, and injects them with saline or norepinephrine [14]. After that, scientists would measure the glucose tolerance test and the C-peptide levels to check the subject's response to saline and norepinephrine [14]. The goal is to determine if short-term elevation of norepinephrine would affect insulin secretion and glucose disposal in subjects with non-insulin-dependent diabetes mellitus (NIDDM) [14]. These results show that the injection of norepinephrine has caused a significant reduction in glucose disposal, no effect on insulin sensitivity, a decrease in insulin secretion rate, and an increase in glucose effectiveness index [14]. The results indicate that in contrast to the control subjects who received the saline injection, there is a physiological elevation of norepinephrine in NIDDM, but it does not reduce or increase insulin sensitivity [14]. Instead, this situation has caused a decrease in the glucose disposal that is related to halting the insulin secretion, which is therefore compensated by increasing the glucose effectiveness index [14]. Despite the accurate and thorough results, there are some critical issues with the research. Since it was conducted in 1997, the results and information are not current enough, and the sample size of eight is too small. The study would be more beneficial if scientists utilize a larger sample size and to make the analysis more current. With these changes implemented, the results and data would be more accurate and meaningful.

 Relationships of depression, anxiety, and stress with adherence to self-management behaviors and diabetes measures in African-American adults with type 2 diabetes

This research was conducted in 2018. Medical scientists would obtain the BMI and glycosylated hemoglobin levels from the medical records of 42 African-American adults with type II diabetes [15]. These subjects were selected on an outpatient basis that is located in an urbanized location. The average age of the participants is around 54.9 years old, and the majority of the subjects are female [15]. Based on the research, the participants will complete a demographic survey and depression, anxiety, and stress scale [15]. The purpose of the study is to check if anxiety, stress, or depression has any effects on the adherence to lifestyle modification such as diet, physical activity, and medication.

**Figure 1:**
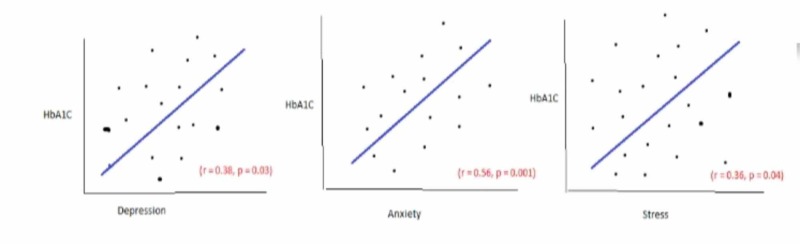
Correlation between depression and HbA1c (glycosylated hemoglobin).

According to Figure 1, we see that the P values for depression, anxiety, and stress are less than 0.05. This data indicates that there is a statistical significance to the result [15]. Plus, the R-value shows a positive correlation between depression, anxiety, and stress to the adherence of lifestyle modifications [15]. If we carefully analyze the data, we can see that anxiety has the strongest correlation to glycosylated hemoglobin levels followed by depression and stress assessment [15]. Basically, individuals who suffer from any of the mental issues often do not adhere to their lifestyle modification regime. Therefore would experience an increase in their glycosylated hemoglobin level which will put them at risk for type II diabetes.

The main issue with the research is that all the patients are in an outpatient clinic and they are limited to African-Americans. The data from other settings in the community, in addition to inpatients and outpatients, should have been explored to have a better understanding to support the conclusion [16]. That way, we could accurately depict the connections between stress, depression, and anxiety with their glycosylated hemoglobin level. This strategy can help determine if other external factors like race and inpatient settings could affect adherence to lifestyle modification. For example, participants from Asian ethnicity may adhere to healthy living despite the stress, anxiety, and depression; hence, they may experience a lower glycosylated hemoglobin level. It is best to eliminate these confounding factors that could affect the experiment [17]. Another limitation is the fact that surveys were used to record the stress, depression, and anxiety level. Keep in mind that surveys can introduce bias because not everybody is honest while they take it [18]. For example, a participant could feel embarrassed about his living situation and adherence to lifestyle modification that he could skew his data to make it look like he is managing everything very well. Therefore, it is best not to use survey as a measuring tool. Instead, scientists should utilize other ways of measuring stress, depression, and anxiety level of the participant. For example, they can have a psychologist to interview these participants to categorize their level of stress, depression, and anxiety. Therefore, with this type of data measurements, you would eliminate these personal biases.

Chronic stress exposure may affect the brain's response to high calorie food cues and predispose to obesogenic eating

In 2013, scientists decided to collect a group of female participants and divided them into two groups [19]. One group of women would be categorized as high chronic stress (HCS) group while the other is placed in low chronic stress (LCS) [19]. Then scientist would utilize an MRI machine for observing the brain activity of these women as they stare at a picture of a high caloric type of food [19]. The primary brain structures that the scientist will measure are the amygdala which depicts emotions, Putamen which focuses on preparation and execution, and the anterior cingulate and anterior prefrontal cortex which emphasize logic and strategy [19]. 

**Figure 2:**
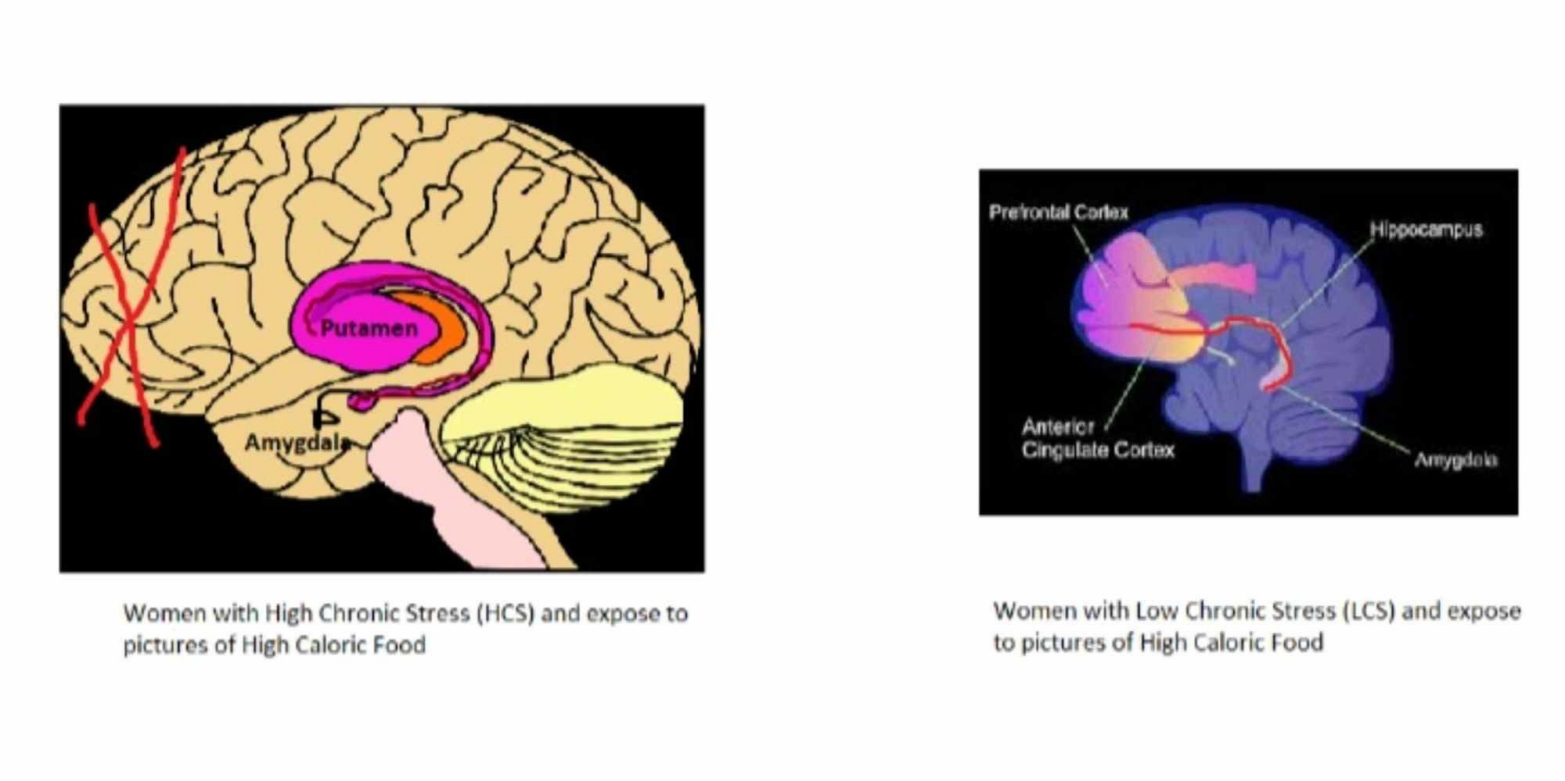
Brain structure between women with high chronic stress (HCS) and women with low chronic stress (LCS). On the left, you will see the brain structure of women with HCS and their exposure to pictures of high caloric food. On the right, you will notice a brain structure of women with LCS and their exposure to pictures of high caloric food. Image taken from Refs. [20-21].

Figure 2 [20-21]-based on the results, women with HCS tend to have a different response than women with LCS when they observe a picture of a hamburger or anything with high calorie [19]. The MRI detects that the women with HCS would experience a deactivation of the frontal region of the brain and would have an enhanced connectivity between the amygdala and Putamen [22]. This makes sense because the frontal cortex involves problem-solving and thinking logically, but that gets shut off. That means the participant in HCS would not be able to rationally believe that the consumption of high-calorie food can affect their health [22]. Instead, they would use their emotions which entail mood and feelings to analyze the situation [19]. Their mood gets elevated, and their emotions are more enhanced when they see a delicious meal in front of them. That is why the amygdala would increase, plus the participant is more likely to react and want a hamburger. Therefore the connectivity between the amygdala and the Putamen would elevate [22]. This is mainly because the Putamen is responsible for preparation and execution. Women with LCS, on the other hand, would have a different reaction towards a picture of high caloric food. Women with a lower stress level would have an increased conductivity between the amygdala and the anterior cingulate and anterior prefrontal cortex. This means that the women in the LCS acknowledges that a high-calorie food like a hamburger is delicious and would make them more satisfied if they consume it, but they can logically reason that such food is unhealthy for them. They would most likely smile and ignore the food to choose something much healthier.

These results indicate that high stress exposure can alter the brain’s response to the way we perceive food. It can predispose the individual to eat more high-calorie food, which can eventually increase the risk of developing obesity and type II diabetes. The main issue with this research is that all the participants are female. Keep in mind that the brain structures between male and female are entirely different [23]. In the future, it is best to obtain both male and female participation so we can observe both brain structures to compare and contrast. This strategy will allow us to see if there are any structural and chemical differences between a man and a woman when they observe a picture of a high-calorie food under various levels of stress.

Take home message

Keep in mind that stress and anxiety can exacerbate type II diabetes but does not cause it. Our research does not indicate a causal effect because some people develop anorexia nervosa under stressful conditions [24]. Anorexia nervosa is an eating disorder where the individual avoids consuming food, therefore, would have lower than average BMI, which raises a question as to why some individuals do not eat under stress [25]? The "flight and fight" response may play a huge role in understanding the concept. When individuals go through a phenomenal stressful situation where their adrenalin is always active or releasing sympathetic hormones, their appetite will inevitably be suppressed [26]. For example, individuals who have gone through an extremely high-stress situation like rape victims may end up developing anorexia nervosa from continuously suppressing their appetite from extreme anxiousness and stress from the situation. However, individuals with ordinary high or moderate stress level such as trying to meet a deadline might end up consuming more food to provide comfort and endorphins to their system to alleviate the current situation [27]. The issue with these analyses is that they do not take into account the accurate way of measuring the level of stress and anxiety in these participants. Despite these issues, individuals with certain stress levels and anxiety do experience a decrease in insulin release; therefore, it reduces the disposal of glucose. It can also make the individual prone to poor lifestyle management and stimulates food cravings. All of which can exacerbate type II diabetes.

## Conclusions

The primary focus of this research article is to find out if stress and anxiety which causes the release of adrenergic and catecholamine hormone would affect the insulin sensitivity, insulin release, and glucose levels. The research emphasizes on non-insulin-dependent diabetic participant to see if stress and anxiety would exacerbate their conditions. We choose these participants mainly because type II diabetes is developed through bad eating habits and low activity levels through everyday life. We want to see if chronic stress can affect lifestyle decisions, and if it plays a role in negatively affecting the physiological activities of the pancreas. We examined and reviewed three different researches in the past that involves stress levels and type II diabetes. With our first research, we found that participants with the norepinephrine injection have experienced a reduction in glucose disposal, decreased insulin secretion rates, increased glucose effectiveness index, and no effect on insulin sensitivity. Our second research shows that individuals with depression, anxiety, and stress have a high correlation to nonadherence to lifestyle modifications. The third research reveals that women in the HCS group would have a strong connection between the Putamen and amygdala, but the frontal cortex is shut off. Women in the LCS group, on the other hand, would have a strong relationship between the prefrontal cortex and the amygdala. The goal of these studies is to provide a deeper insight to healthcare providers on managing diabetic patients. A lot of times, doctors believe that type II diabetes has a lot to do with medication noncompliance and failure to adhere to lifestyle modification. Unfortunately, they fail to realize that stress and anxiety, as well as other mental problems, can also exacerbate the rising glucose level. With this in mind, doctors can better consult their patients by advising them to consider stress reduction exercises like yoga, meditation, or seeing a counselor to help reduce their stress and anxiety levels. 
